# *Population Health Metrics*: the next phase

**DOI:** 10.1186/s12963-020-0202-z

**Published:** 2020-02-05

**Authors:** Jonathan M. Samet, Christopher J. L. Murray, Alan D. Lopez

**Affiliations:** 1 Colorado School of Public Health, Aurora, CO USA; 2 Institute for Health Metrics and Evaluation, Seattle, WA USA; 3 University of Melbourne, Melbourne, Australia

First published in 2003, Population Health Metrics has evolved into a leading international journal for those concerned with how to measure the health of populations and to assess the consequences of interventions to improve population health. Interest in these broad methodological topics has grown substantially since the journal was launched as goals have been set for advancing health, e.g., the Sustainable Development Goals, and increasing calls by the global health community for accountability as to what has been accomplished by programs and policies that have been implemented and by funds expended. Over the years, since the journal was founded, the term “population health” has become ever more widespread in its usage, now extending to the health care domain in the USA, for example. The term has the advantage of an implied inclusive breadth that extends beyond the concept of “public health”.

That breadth is illustrated by the range of papers published by *Population Health Metrics*. The journal has given emphasis to novel methods for measuring and tracking population health, while addressing such topics as tobacco use, air pollution, verbal autopsies, infectious diseases and, non-communicable diseases. It stands out for bringing together papers on innovative methods with papers showing how such methods deepen understanding of population health and its determinants.

That breadth is further exemplified by considering some of the journal’s most cited papers (Table 1).

**Table 1:** *Population Health Metrics*’ most cited papers

Article title	Corresponding author	Year	DOI	Number of citations
Projection of the year 2050 burden of diabetes in the US adult population: dynamic modeling of incidence, mortality, and prediabetes prevalence	James P Boyle	2010	10.1186/1478-7954-8-29	746
National, regional, and global trends in adult overweight and obesity prevalences	Gretchen A Stevens	2012	10.1186/1478-7954-10-22	449
Accuracy and completeness of mortality data in the Department of Veterans Affairs	Min-Woong Sohn	2006	10.1186/1478-7954-4-2	280
A generic model for the assessment of disease epidemiology: the computational basis of DisMod II	Jan J Barendregt	2003	10.1186/1478-7954-1-4	211
Comparative quantification of health risks: conceptual framework and methodological issues	Christopher JL Murray	2003	10.1186/1478-7954-1-1	209
The burden of disease and injury in Iran 2003	Mohsen Naghavi	2009	10.1186/1478-7954-7-9	192
Mortality registration and surveillance in China: history, current situation and challenges	Gonghuan Yang	2005	10.1186/1478-7954-3-3	167
Global epidemiology of invasive meningococcal disease	Rabab Z Jafri	2013	10.1186/1478-7954-11-17	166
Algorithms for enhancing public health utility of national causes-of-death data	Mohsen Naghavi	2010	10.1186/1478-7954-8-9	163
Modeling causes of death: an integrated approach using CODEm	Kyle J Foreman	2012	10.1186/1478-7954-10-1	162

The journal is publishing around 20–30 papers per year. The 2-year Impact Factor has remained comparatively steady around 2.8/2.9, whilst the 5-year Impact Factor is currently 3.616. This figure ranks the journal as one of the top 5 Open Access journals within the SSCI category ‘Public, Environmental & Occupational Health’.

In 2017, BMC signed the San Francisco Declaration on Research Assessment (DORA) and as part of this, *Population Health Metrics* committed to presenting the Impact Factor in the context of a variety of journal-based metrics. These measures include the 2-year Impact Factor, 5-year Impact Factor, SCImago Journal Rank, Source Normalized Impact per Paper (SNIP), article downloads, and article handling times. All of these metrics are openly available on the journal homepage.

This status report comes as the team of editors for *Population Health Metrics* changes. The founding Editors-in-Chief, Christopher JL Murray and Alan D Lopez stepped down in 2019 and are replaced by Jonathan M. Samet. A new team of Associate Editors and Editorial Board members is being recruited. The Scope and Aims of *Population Health Metrics* leaves its broad mission unchanged:

“*Population Health Metrics* aims to advance the science of population health assessment, and welcomes papers relating to concepts, methods, ethics, applications, and summary measures of population health.”

The journal provides a unique platform for population health researchers to share their findings with the global community. We seek research that addresses the communication of population health measures and policy implications to stakeholders; this includes papers related to burden estimation and risk assessment, and research addressing population health across the full range of development.

*Population Health Metrics* covers a broad range of topics encompassing health state measurement and valuation, summary measures of population health, descriptive epidemiology at the population level, burden of disease and injury analysis, disease and risk factor modeling for populations, and comparative assessment of risks to health at the population level. The journal is also interested in how to use and communicate indicators of population health to reduce disease burden, and the approaches for translating from indicators of population health to health-advancing actions. As a cross-cutting topic of importance, we are particularly interested in inequalities in population health and their measurement.”

As a foundational principle, *Population Health Metrics* proposes that the ultimate goal of all of its papers should be to advance population health, both locally and globally. Looking to the future of population health and the journal’s role, an increasing emphasis on studies addressing the consequences of policies, however enacted, can be anticipated. Tools for estimating burden have been refined and yielded results that are trusted and that have motivated action. The call for documentation of benefits of actions is ever louder, but providing answers can be challenging.

At this time of transition, the new editorial team thanks and acknowledges those who launched the journal, particularly Alan Lopez and Chris Murray, and the numerous contributors who served as Associate Editors, Editorial Board members, and reviewers.

